# Magnitude and associated factors of anemia among AZT based HAART experienced adult HIV patients at University of Gondar Comprehensive Specialized Referral Hospital, Northwest, Ethiopia, 2019: a retrospective cohort study

**DOI:** 10.1186/s12879-021-06712-5

**Published:** 2021-09-28

**Authors:** Zegeye Getaneh, Worku Wale, Belete Chanie, Etetetu Temesgen, Metadele Abebe, Melesse Walie, Mulualem Lemma

**Affiliations:** 1 Department of Hematology and Immunohematology, School of Biomedical and Laboratory Sciences, College of Medicine and Health Sciences, University of Gondar, P.O. Box 196, Gondar, Ethiopia; 2 School of Biomedical and Laboratory Sciences, College of Medicine and Health Sciences, University of Gondar Hospital, Gondar, Ethiopia; 3 Department of Immunology and Molecular Biology, School of Biomedical and Laboratory Sciences, College of Medicine and Health Sciences, University of Gondar, Gondar, Ethiopia

**Keywords:** Anemia, Hemoglobin, Zidovudine, HIV/AIDS, Associated factors

## Abstract

**Background:**

Anemia is the most common hematologic abnormalities in AIDS patients usually associated with disease progression and poor clinical outcomes. Zidovudine (AZT), which is one of the nucleoside reverse transcriptase inhibitor drug families of the first line antiretroviral therapy regimen for HIV/AIDS patients, causes anemia due to early long-term of higher-dose therapy. This study was aimed to assess the magnitude and associated factors of anemia among AZT containing HAART experienced adult HIV/ADIS patients at University of Gondar Comprehensive Specialized Referral Hospital, northwest, Ethiopia, 2019.

**Methods:**

A retrospective cohort study was conducted among a total of 320 adult AZT based HAART experienced HIV/AIDS patients from January 2016 to December 2018. Systematic random sampling technique was used to select the patients’ charts. All required data for this study were extracted from patients’ medical charts. Data were coded, cleared and entered into Epi Info version 3.5.3, and transformed to SPSS version 20 for analysis. Descriptive statistics, bivariable and multivariable logistic regression models were fitted to identify associated factors of anemia and P-value < 0.05 was considered as statistically significance.

**Results:**

A total of 320 adult AZT based HAART experienced HIV/AIDS patients’ charts were assessed. Of the total patients, 198 (61.9%) were females and 133 (41.6%) were within the age range of 35–45 years. More than half, 237(76.9%) of the patients were from the urban area and 186 (58.1%) were on WHO clinical stage III at the baseline. The prevalence of anemia was 50% (95% CI 44.7–55.0%), 44.1% (95% CI 38.4–50.0%), 35.6% (95% CI 30.3–40.6%), 40% (95% CI 34.4–45.6%), 40.6% (95% CI 35.0–46.3) and 39.1% (95% CI 33.4–44.1%) at baseline, 6 months, 12 months, 18 months, 24 months and 30 months of follow-up period, respectively. The overall prevalence of anemia was 41.6%. Anemia had significant association with WHO clinical stage and base line Hgb values.

**Conclusions:**

A significant number of participants were anemic in this study. WHO clinical stage and baseline Hgb value were the contributing factors for anemia among these patients. Therefore, anemia needs an immediate intervention on associated factor to improve the anemic status and living condition of HIV patient.

**Supplementary Information:**

The online version contains supplementary material available at 10.1186/s12879-021-06712-5.

## Background

The utilization of combined antiretroviral therapy (ART) was useful to alleviate the burden of human immunodeficiency virus (HIV) pandemic in resource restricted settings including Ethiopia. Combination ART decreases the mortality rate, progression of HIV to AIDS, expedient infections, and hospitalization due to HIV viral suppression [1]. In Ethiopia, many thousands of ART indigent individuals living with HIV are registered in ART because of the improved coverage of free antiretroviral medical aid program [2]. Per the revised 2018 national comprehensive HIV care and treatment guideline, the sequencing choices for most popular first, second and third-line ART regimens for adults and adolescents are: TDF + 3TC + DTG or TDF + 3TC + EFV, AZT + 3TC + ATV/r or LPV/r, and DRV/r + ABC + 3TC + EFVor NVP, respectively. Whereas for those pregnant, breastfeeding and childbearing age women, AZT + 3TC + EFV/NVP, TDF + 3TC + ATV/r or LPV/r, and DRV/r + DTG + TDF + 3TC are the most popular first, second and third-line ART regimens, respectively. Whereas TDF + 3TC + EFV/NVP, AZT + 3TC + ATV/r or LPV/r and DRV/r + DTG + TDF + 3TC are the second alternatives for first, second and third-line ART regimens, respectively. The last option is ABC + 3TC + EFV/NVP, AZT + 3TC + ATV/r or LPV/r and DRV/r + ABC + 3TC + EFVor NVP, correspondingly [3]. However, failure of ART and toxicity became the alarming drawback of long-run ART users [4].

Human immunodeficiency virus (HIV) infection is related to profound hematological abnormalities within which anemia being the key one often discovered among these patients [5]. Anemia has been estimated to vary from 30 to 95% among these patients [6, 7]. In the early 1990s, the prevalence of anemia in HIV-positive patients reached 90%, then with the widespread adoption of highly active antiretroviral therapy (HAART) it became decreased to approximately 46% [8].

Anemia is a major health problem globally, which can cause a vicious impediment on quality of life, mortality, morbidity, and socioeconomic progress of individuals especially in the developing countries [9]. Anemia has been associated with disease progression and poor clinical outcomes in HIV/AIDS patients [10].

Even though the problem of anemia was no longer thoroughly understood, it become expected that approximately60 to 80% of HIV/AIDS individuals sufferers of their late-degree of the sickness had been affected globally. Of these patients, around 22% is considered to be associated with the treatment regimens including ART provided to the patients. Moreover, it had been observed that females have been strangely more tormented by the situation in comparison to males. For example, in the US up to 71% extra incidence of anemia had been recorded among women than men [11].

The prevalence of anemia became increasing with disease advancement. For example, about 28% of HIV infected individuals in the pre AIDS stage of the disease develop anemia, whereas it could reach as high as 71% in the advanced or AIDS stage of the HIV disease [12]. On the other hand, anemia ranges from 23 to 58% in adult HIV patients on ART [13].

Anemia is a widely spread hematological abnormality in HIV/AIDS patients in developing countries. For example, 54% [14] and 60.6% [15] of HIV infected individuals were anemic in Egypt and Nigeria, respectively. Likewise, large segments of HIV infected patients have been suffering from anemia and anemia related complications in Ethiopia. For example, in Addis Ababa, 52.6% of HIV infected patients on ART were anemic in 2012 [16].

The causes of anemia in HIV infected individuals are multifactorial in origin. Infiltrations of the bone marrow by neoplasm or infection, use of myelosuppressive medications, decrease production of endogenous erythropoietin, hemolysis due to RBC autoantibodies and HIV infection itself are some of them [17–23].

On the other hand, nutritional deficiencies; most commonly, deficiencies in iron, folic acid, or vitamin B12 are also be causes of anemia. In patients with HIV disease, folic acid deficiency is generally caused by either dietary deficiency or jejunal pathology [23]. Vitamin B12 deficiency may result from malabsorption in the ileum or from gastric pathology caused by an array of infections or other conditions that affect the gastric mucosa in HIV-infected patients [24]. HIV medications contains zidovudine (AZT) and other drugs are considered as the causes of anemia in HIV/AIDS patients [8, 25].

Zidovudine (AZT) is one of the nucleoside reverse transcriptase inhibitor drug families used together with other ART drugs to slow down the progression of HIV infection. Due to delay progression of infection, the immune system become improving which is characterized by an increase in helper lymphocyte cell count [26].

However, AZT is known to be associated with life threatening hematological toxicity like anemia due to early long-term of higher-dose therapy. AZT also causes bone marrow suppression, which causes anemia. Bone marrow toxicity appears to be more common in those patients with advanced disease and related to dose and duration of the treatment. All of these conditions were generally found to be reversible upon reduction of AZT dosages. They have been attributed to several possible causes, including transient depletion of mitochondrial DNA, sensitivity of the DNA polymerase in some cell mitochondria [27]. The depletion of thymidine triphosphate, oxidative stress, and reduction of intracellular l-carnitine or apoptosis of the muscle cells [28].

AZT is associated with a risk of hematological toxicity, and measuring hemoglobin is recommended before initiating ART and monthly at least for the next three months. Avoid use of AZT as first-line therapy for people with HIV if there is severe anemia at baseline (hemoglobin < 7.0 g/dl) [3]. AZT is known to be associated with myelotoxity that often presents with anemia. However, the magnitude of anemia and its associated risk factors among HIV patients on AZT based HAART are not well document in Ethiopia. Therefore, this study aimed to determine the prevalence and associated factors of anemia among adult HIV/AIDS infected patients on AZT containing HAART at the University of Gondar Comprehensive Specialized Referral Hospital, northwest Ethiopia.

## Methods

### Study design and setting

A retrospective cohort study was conducted at the University of Gondar specialized referral hospital ART clinic. The hospital is located in Gondar town, Central Gondar Zone, Amhara regional state, Ethiopia. Gondar town is located 727 km away from Addis Ababa, the capital city of Ethiopia and 180 km far from Bahir Dar city, the capital city of Amhara regional state in northwest direction. The town is situated at a latitude and longitude of 12°36’N 37°28’E with an elevation of 2133 m above sea level [29]. Currently, the hospital has been serving people from Central, North and Western Gondar Zone as well as the surrounding districts of Benshangul Region. Around 5 million dwellers of the North, West and Central Gondar Zone, and those of the neighboring district of Northwest Ethiopia have been seek medical services, including HIV care and treatment in this hospital. As of 2018, there had been almost 10,000 HIV/AIDS patients with ongoing follow-up in the ART clinic of the hospital, of which 5600 had been on combination AZT based HAART. This study was performed from February 01 to May 30, 2019.

### Study population and sampling

A total of 320 AZT based HAART experienced adults HIV/AIDS patients’ medical charts had been selected by a systematic random sampling approach and all of the essential data were collected. The single population proportion formula was used to determine the sample size by considering the formerly reported 25% prevalence of anemia [30], with 95% confidence interval (CI) and 5% margin of error. All adult HIV infected patients greater than or equal to 18 years old who have been taking AZT based HAART drugs in the clinic from January 2016 to December 2018 have been taken into consideration for the study population. Totally 5600 AZT based HAART experienced HIV/AIDS patients have been served in the ART clinic during the course of the study period. The participants’ medical charts were selected at approximate sampling interval of 18 (5600/320) from their sequence of ART clinic visit. The first participant of the study was chosen by lottery method from the 18 order of adult HIV/AIDS patients. Afterwards, participants were enrolled in the study at every 18th interval till the overall sample size become achieved.

### Inclusion and exclusion criteria

All adult HIV/AIDS patients aged greater than or equal to 18 years who were taking AZT based HAART drugs in the hospital from January, 2016 to December, 2018 were included in the study. Patients with incomplete record at base line and follow up, pregnant patients, patients with chronic kidney disease, rheumatic arthritis, cancer, tuberculosis, liver disease, diabetics, cardiovascular disease, burns and gastrointestinal diseases were excluded from the study.

### Definitions of terms

The study outcome variables were defined based on World Health Organization (WHO) criteria [31]: anemia in men was defined as an adjusted Hgb levels lower than 13 g/dL (11–12.9 g/dL, 8–10.9 g/dl and < 8 g/dL for mild, moderate and severe anemia, respectively) and while it was 12 g/dL (10–11.9 g/dL, 8–10.9 g/dL and < 8 g/dL for mild, moderate and severe anemia, respectively) for adult non-pregnant women.

### Data collection tool and process

First patients’ charts were identified and retrieved using the patient registration number found in the registration log book. Then appropriate data extraction format (check list) was developed based on the objective of the study and pretested. All the available data on the patient’s charts had been identified and those valuable once for the study had been collected by three nurses. The pretested data collection format contains sociodemographic data and clinical information such as unique ART numbers, date when treatment started, baseline CD-4 count, WHO clinical stage, ART drug combination, body mass index (BMI), opportunistic infections (OPIs), baseline and current Hgb levels, and AZT based HAART regimen.

### Data quality control and management

The limpidity and completeness of the data collection format was pre-tested on randomly selected patients’ charts before the actual data collection had been started. Corrections and modification were done on the data collection format based on available data and review of previous articles. Completed data collection tools were checked regularly for completeness of information. Intensive training had been given to the data collectors regarding the objective of the study and how to extract or collect data from patients’ charts. Additionally, data collection was closely supervised.

### Data processing and analysis

After data were checked and entered in to EPI Info version 3.5.3 statistical software, verification were made and then they had been exported to Statistical Package for Social Sciences (SPSS) version 20 for analysis. Data cleaning was performed to check for frequencies, accuracy, consistencies and missed values. Descriptive statistics of all the relevant data like sociodemographic variables, baseline CD-4 count, WHO clinical stage, BMI, OPIs, baseline and current Hgb levels, and AZT based HAART regimen were done and presented by tables. A binary logistic regression analysis and Generalized Estimating Equations (GEE) model have been used to fit a repeated measures logistic regression analysis to study the effect of AZT based HAART on patients and to determine the level of association between the independent variables and anemia. All the explanatory variables were analyzed by bivariable logistic regression model by the enter method. Then, those variables with P-value < 0.2 in the bivariable logistic regression model were fitted into multivariable logistic regression model and analysed by the backward LR to identify independently associated factors with anemia. The strength of statistical association was measured by adjusted odds ratios (AOR) and 95% confidence intervals (CI). P-value < 0.05 was considered as statistical significance. The incident outcome-survival analysis approach using a Kaplan–Meier curve was used to show time to anemia in the cohort, as well as by important factors such as WHO stage at baseline, binary and adjusted Cox regression model.

## Results

### Baseline characteristics of the study participants

A total of 320 adult AZT based HARRT experienced HIV/AIDS patients’ charts were assessed and all the valuable data were collected for this retrospective study. The majority, 198 (61.9%) of the study participants were females. Around 133 (41.6%) were within the age range of 35–45 years. Most, 237 (76.9%) of the patients were urban inhabitants and 186 (58.1%) of the patients were at WHO clinical stage III but only 66 (20.1%) were underweight. All most all of the patients, 297 (92.8%) had CD4 count greater than 200 cell/mm^3^. The mean Hgb value was 12.3 g/dl with 1.7 g/dl standard deviation (SD) whereas the mean CD4 cell count was 348.2 (SD = 223.9) cells/mm^3^ (Table 1).

**Table 1:** Baseline socio-demographic and clinical characteristics of HIV infected adult patients on AZT based HAART at University of Gondar Specialized Referral Hospital ART clinic, Gondar, Ethiopia, 2016–2018 (n = 320)

Variable	Category	Frequency	Percentage
Age	< 35 years	110	34.4
	35–45 years	133	41.6
	45 years	77	24.1
Sex	Male	122	38.1
	Female	198	61.9
Residence	Urban	237	74.1
	Rural	83	25.9
Educational status	No formal education	49	15.3
	Primary education	88	27.5
	Secondary	131	40.9
	Tertiary	52	16.3
Baseline WHO clinical stage	I	41	12.8
	II	56	17.5
	III/IV	223	69.7
opportunistic infection	Yes	42	13.1
	No	278	86.9
Baseline CD 4 count	< 200 cell/μl	80	25
	> 200 cell/μl	240	75
Social drug use	Yes	20	6.2
	No	300	93.8
Baseline anemia status	Anemic	160	50
	Non-anemic	160	50
Baseline BMI	Under weight	97	30.3
	Normal	171	53.4
	Over weight	52	16.3

### Anemia before AZT based HAART initiation

A total of 160 (50%) patients were anemic at baseline of which 95 (29.7%), 61 (19.1%) and 4 (1.3%) had mild, moderate and severe anemia, respectively. The proportion of anemia was higher in females (52.5%) than in males (47.5%) (*P* < 0.001). Higher proportion of females had moderate and severe forms of anemia: moderate anemia was 39 (46.4%) among females and 22 (28.9%) among males and severe anemia 4 (4.8%) among females. About 54 (71.1%) of the anemic males and 41 (48.8%) of anemic females were mildly anemic at the baseline. The types of anemia observed before the initiation of HAART were 14 (8.8%), 52 (32.5%), 110 (68.5%) microcytic hypochromic, normocytic normochromic, and macrocytic normochromic anemia, respectively.

Different factors were observed to be associated with being anemic before the initiation of AZT based HAART. In the univariable logistic regression analysis; sex, age, residence, OPIs, BMI, CD4 count, educational status and WHO clinical stage were analyzed. Except residence and OPIs, all the variables having a p-value of less than or equals to 0.2 in univariable logistic regression analysis had been fitted to the multivariable logistic regression model. In multivariable logistic regression analysis, male sex (AOR 2.63; 95% CI 1.59, 4.35), younger age (< 35 years) (AOR 2.083; 95% CI 1.096, 3.961), WHO clinical stages; stage II (AOR 3.024; 95% CI 1.265, 7.228) and stage III/IV (AOR 2.176; 95% CI 1.054, 4.491) were independently associated with increased risk of anemia at baseline with a P-value of less than 0.05 (Table 2).

**Table 2:** Predictors of anemia in HIV infected patients before AZT based HAART initiation at University of Gondar Specialized Referral Hospital ART clinic, Gondar, Ethiopia, 2016 (n = 320)

Variables	Category	Baseline Anemia status		Univariable analysis		Multivariable analysis	
		Anemic	Non-anemic	*P-value*	COR (95% CI)	*P-value*	AOR (95% CI)
Age of ART initiation	< 35 years	59 (53.6%)	51 (46.4%)	0.203	1.463 (0.815, 2.227)	0.025	2.083 (1.096, 3.961)
	35–45 years	67 (50.4%)	66 (49.6%)	0.385	1.284 (0.731, 2.256)	0.194	1.553 (0.854, 2.823)
	> 45 years	34 (44.2%)	43 (55.8%)	1			
Sex	Male	76 (62.3%)	46 (37.7%)	0.001	2.24 (1.41, 3.56)	0.000	2.63 (1.59, 4.35)
	Female	84 (42.4%)	114 (57.6%)	1			
Residence	Urban	114 (48.1%)	37 (44.6%)	1			
	Rural	46 (55.4%)	123 (51.9%)	0.253	1.341 (0.812, 0.217)		
Educational status	No	25 (51.0%)	24 (49.0%)	0.501	0.764 (0.348, 1.675)	0.526	0.760 (0.325, 1.777)
	Primary	47 (53.4%)	41 (46.6%)	0.623	0.841 (0.421, 1.678)	0.340	0.693(0.326, 1.471)
	Secondary	58 (44.3%)	73 (55.7%)	0.102	0.583 (0.304, 1.115)	0.128	0.581(0.289, 1.169)
	Tertiary	30 (57.7%)	22 (42.3%)	1			
WHO clinical stage	Stage I	14 (34.1%)	27 (65.9%)	1			
	Stage II	32 (57.1%)	24 (42.9%)	0.027	2.571 (1.116, 5.925)	0.013	3.024 (1.265, 7.228)
	Stage III/IV	114 (51.1%)	109 (48.9%)	0.048	2.017 (1.005, 4.049)	0.036	2.176 (1.054, 4.491)
Baseline CD 4 count	< 200 cell/μl	47 (58.8%)	33 (41.2%)	0.072	1.601 (0.959, 2.672)	0.082	1.620 (0.941, 2.789)
	> 200 cell/μl	113 (47.1%)	127(52.9%)	1			
BMI at baseline	Under weight	56 (57.7%)	41(42.3%)	0.178	1.593 (0.809, 3.139)	0.298	1.462 (0.715, 2.991)
	Normal	80 (46.8%)	91(53.2%)	0.936	1.026 (0.550, 1.911)	0.911	0.964 (0.503, 1.848)
	Over weight	24 (46.2%)	28 (53.8%)	1			
Opportunistic infection	Yes	22 (52.4%)	20 (47.6%)	0.741	1.116 (0.583, 2.137)		
	No	138 (49.6%)	140 (50.4%)	1			

*COR* crudes odds ratio, *AOR* adjusted odds ratio

### Anemia after AZT based HAART initiation

A total of 320 AZT based HAART experienced adult HIV patients were enrolled and all had a Hgb measurement at 6, 12, 18, 24 and 30 months of HAART initiation. After the initiation of AZT based HAART, 141 (44.1%), 114 (35.6%), 128 (40%), 130 (40.6%) and 125 (39.1%) of the participants were anemic. The prevalence of anemia was 44.1% (95% CI 38.4, 50.0%), 35.6% (95% CI 30.3, 40.6%), 40% (95% CI 34.4, 45.6%), 40.6% (95% CI 35.0, 46.3) and 39.1% (95% CI 33.4, 44.1%), 6 months, 12 months, 18 months, 24 months and 30 months after AZT based ART treatment, respectively. Greater than 41% of anemic patients were within the age range of 35–45 years. Majority 20% of patients who developed anemia had a baseline Hgb level of ≤ 12 g/dl. From the total anemic participants, 57.8%, 70.3%, 22.2% and 66.8% were females, urban residents, have CD4 count less than 200 cells/mm^3^, and on AZT + 3TC + NVP combination therapy, respectively. Additionally, of the total anemic cases, 10.7%, 18.3% and 71.1% were at WHO clinical AIDS stage I, stage II, and stage III/IV. The prevalence of mild, moderate and severe anemia at the baseline were 95 (29.7%), 61 (19.1%) and 4 (1.3%), respectively. Whereas the prevalence of mild, moderate and severe anemia at the 30 months of treatment were 82 (25.6%), 41 (12.8%) and 2 (0.6%), respectively (Table 3).

**Table 3:** Magnitude and characteristics of anemia among adult patients taking AZT based HAART treatment at University of Gondar Specialized Referral Hospital ART clinic from January 2016—February 2018

Variables	Category	Anemia status										
		6 month		12 month		18 month		24 month		30 month		
		Yes N (%)	No N (%)	Yes N (%)	No N (%)	Yes N (%)	No N (%)	Yes N (%)	No N (%)	Yes N (%)	No N (%)	
Age of ART initiation	< 35 years	52 (47)	58 (53)	36 (33)	74 (67)	44 (40)	66 (60)	44 (40)	66 (60)	43 (39)	67 (61)	
	35–45 years	54 (41)	79 (59)	49(37)	84 (63)	51 (38)	82 (62)	57 (43)	76 (57)	54 (41)	79 (59)	
	> 45 years	35 (46)	42 (54)	29(38)	48 (62)	33 (43)	44 (57)	29 (38)	48 (62)	28 (36)	49 (64)	
Sex	Male	65 (53)	57 (47)	40(33)	82 (67)	53 (43)	69 (57)	58 (48)	64 (52)	45 (37)	77 (63)	
	Female	76 (38)	122 (62)	74(37)	124 (63)	75 (38)	123 (62)	72 (36)	126 (64)	80 (40)	118 (60)	
Residence	Urban	106 (45)	131 (55)	78 (33)	159 (67)	84 (35)	153 (65)	38 (46)	45 (54)	38 (46)	45 (54)	
	Rural	35 (42)	48 (58)	36 (43)	47 (57)	44 (53)	39 (47)	92 (39)	145 (61)	87 (37)	150 (63)	
Educational status	No	24 (49)	25 (51)	16(33)	33 (67)	21 (43)	28 (57)	18 (37)	31 (63)	16 (33)	33 (67)	
	Primary	38 (43)	50 (57)	32(36)	56 (64)	35 (40)	53 (60)	40 (45)	48 (55)	38 (43)	50 (57)	
	Secondary	57 (44)	74 (56)	49(37)	82 (63)	50 (38)	81 (62)	48 (37)	83(63)	52 (40)	79 (60)	
	Tertiary	22 (42)	30 (58)	17(33)	35 (67)	22 (42)	30 (58)	24 (46)	28 (54)	19 (36)	33 (64)	
WHO clinical stage	Stage I	19 (46)	22 (54)	12(29)	29 (71)	13 (32)	28 (68)	14 (34)	27 (66)	13 (32)	28 (68)	
	Stage II	23 (41)	33 (59)	22(39)	34 (61)	20 (36)	36 (64)	24 (43)	32 (57)	25 (45)	31 (55)	
	Stage III/IV	99 (44)	124 (56)	80(36)	143(64)	95 (43)	128(57)	92 (41)	131 (59)	87 (39)	136 (61)	
Baseline CD 4 count	< 200 cell/μl	30 (38)	50 (62)	25(31)	55 (69)	24 (30)	56 70)	30 (38)	50 (62)	21 (26)	59 (74)	
	> 200 cell/μl	111(46)	129 (54)	89(37)	151 (63)	104 (43)	136 (57)	100 (41)	140 (58)	104 (43)	136 (57)	
BMI at baseline	Under weight	47 (48)	50 (52)	37 (38)	60 (62)	39 (40)	58 (60)	43 (44)	54 (56)	44 (45)	53 (55)	
	Normal	70 (41)	101 (59)	61 (36)	110 (64)	68 (40)	103 (60)	70 (41)	101 (59)	56 (33)	115 (67)	
	Over weight	24 (46)	28 (54)	16 (31)	36 (69)	21 (40)	31 (60)	17 (33)	35 (67)	25 (48)	27 (52)	
Opportunistic infection	No	120 (43)	158 (57)	03(37)	175 (63)	116 (63)	162 (58)	103 (39)	171 (61)	109 (39)	169 (61)	
	Yes	21 (50)	21 (50)	11 (26)	31 (74)	12 (29)	30 (71)	23 (55)	19 (45)	16 (38)	26 (62)	
AZT-based HAART regimen	AZT + 3TC + NVP	94 (44)	118 (56)	78 (37)	134 (63)	79 (37)	133 (63)	88 (42)	124 (58)	87 (41)	125 (59)	
	AZT + 3TC + EFV	41 (43)	54 (57)	31 (33)	64 (67)	45 (47)	50 (53)	37 (39)	58 (61)	34 (36)	61 (64)	
	AZT + 3TC + ATV/r	6 (55)	5 (45)	5 (45)	6 (55)	3 (27)	8 (73)	4 (36)	7 (64)	4 (36)	7 (64)	
	AZT + 3TC + NVP/r	0 (0)	2 (100)	0 (0)	2 (100)	1 (50)	1 (50)	1 (50)	1 (50)	0 (0)	2 (100)	

Of the anemic study participants at the baseline, 14 (8.5%), 110 (68.75%) and 36 (22.5%) had microcytic (MCV < 80 fl), macrocytic (MCV > 100 fl) and normocytic (MCV 80–100 fl) type of anemia, respectively. While 6 (4.8%), 112 (89.6%) and 7 (5.6%) anemic patients had microcytic, macrocytic and normocytic types of anemia, respectively at 30 months after the initiation of AZT based HAART. On the other hand, 93 (58.1%), 29 (18.1%) and 38 (23.8%) of the anemic patients had polychromic (MCH > 32 pg), hypochromic (MCH: < 27 pg) and normochromic (MCH: 27–32 pg) types of anemia, respectively at baseline. While 111 (88.8%), 7 (5.6%) and 7 (5.6%) of the anemic patients had polychromic, hypochromic, and normochromic types of anemia, respectively at 30 months after HAART initiation. Around 17 (68.0%) and 21 (16.8%) of the anemic patients had anisocytosis (RDW > 14.5%) RBC population at baseline and after 30 months of HAART initiation, respectively (Table 4).

**Table 4:** Distribution RBC indices among anemic adult patients taking AZT based HAART treatment at University of Gondar ART clinic from January 2016 to February 2018

Variable	Category	Proportions n (%)					
RBC Indices		Baseline N = 160	6 month N = 141	12 month N = 114	18 month N= 128	24 month N = 130	30 month N = 125
MCV	Microcytic: < 80 fl	14 (8.5%)	13 (9.2%)	10 (8.8%)	11 (8.6%)	11 (8.5%)	6 (4.8%)
	Normocytic: 80-100 fl	36 (22.5%)	34 (24.1%)	21 (18.4%)	14 (10.9%)	10 (7.7%)	7 (5.6%)
	Macrocytic: > 100 fl	110 (68.75%)	94 (66.7%)	83 (72.8%)	103 (80.5%)	109 (83.8%)	112 (89.6%)
MCH	Hypochromic: < 27 pg	29 (18.1%)	16 (11.3%)	13 (11.4%)	13 (10.2%)	12 (9.2%)	7 (5.6%)
	Normochromic: 27-32 pg	38 (23.8%)	16 (11.3%)	16 (14.0%)	11 (8.6%)	8 (6.2%)	7 (5.6%)
	Polychromic: > 32 pg	93 (58.1%)	109(77.3%)	85 (74.6%)	104 (81.2%)	110 (84.6%)	111 (88.8%)
MCHC	Hypochromic: < 32 g/dl	73 (45.6%)	40 (28.4%)	34 (29.8%)	26 (20.3%)	39 (30.0%)	23 (18.4%)
	Normochromic:32-36 g/dl	76 (47.5%)	89 (63.1%)	62 (54.4%)	78 60.9%)	74 (56.9%)	83 (66.4%)
	Polychromic: > 36 g/dl	11 (6.9%)	12 (8.5%)	18 (15.8%)	24 (18.8%)	17 (13.1%)	19 (15.2%)
RDW	Low: < 11.5%	43 (50.6%)	26 (18.4%)	19 (16.7%)	23 (18.0%)	23 (17.7%)	24 (19.2%)
	Normal: 11.5–14.5%	100 (47.6%)	105(74.5%)	80 (70.2%)	86 (67.2%)	96 (73.8%)	80 (64.0%)
	High: > 14.5%	17 (68.0%)	10 (7.1%)	15 (13.2%)	19 (14.8%)	11 (8.5%)	21 (16.8%)
Severity of Anemia	Mild: 11–11.9 g/dl	95 (29.7%)	95 (29.7%)	85 (26.6%)	87 (27.2%)	93 (29.1%)	81 (25.3%)
	Moderate: 8–10.9 g/dl	61 (19.1%)	44 (13.8%)	24 (7.5%)	40 (12.5%)	34 (10.6%)	42 (13.1%)
	Severe: < 8 g/dl	4 (1.3%)	2 (0.6%)	5 (1.6%)	1 (0.3)	3 (0.9)	2 (0.6%)

### Factors associated with anemia prevalence

Generalized Estimating Equations (GEE) model have been used to fit a repeated measures logistic regression to study effects of AZT based HAART on adult HIV/AIDS patients. Anemia status was taken as a dependent variable and sex, residence, educational status, baseline WHO clinical stage, social drug use, OPI, ART regimen combination, age category, baseline BMI, baseline CD4 count, and duration of AZT treatment were taken as independent or intercept model (Table 5 and Additional file 1).

**Table 5:** Categorical variable information and parameter estimates of the GEE model analysis of adult patients taking AZT based HAART treatment at University of Gondar Specialized Referral Hospital ART clinic from January 2016, to February 2018

Variables	Category	Overall (N = 1920)	Anemia status		P-value	Exp(B)	95% Wald CI for Exp (B)	
		N (%)	Anemic N = 798 (41.6%)	Non-anemic N = 1122(58.4%)	Sign	AOR	Lower	Upper
Age of ART initiation	< 35 years	660 (34.4%)	278 (42.1%)	382 (57.9%)	0.406	1.202	0.778	1.858
	35–45 years	798 (41.6%)	332 (41.6%)	466 (58.4%)	0.549	1.136	0.749	1.723
	> 45 years	462 (24.1%)	188 (40.7%)	274 (59.3%)	–	1	–	–
Sex	Male	732 (38.1%)	337 (46.0%)	395 (54.0%)	0.011	1.527	1.102	2.114
	Female	1188 (61.9%)	461 (38.8%)	727 (61.2%)	–	1	–	–
Residence	Urban	1422 (74.1%)	561 (39.5%)	861 (60.5%	–	1	–	–
	Rural	498 (25.9%)	237 (47.6%)	261 (52.4%)	0.056	1.388	0.992	1.943
Educational status	No	294 (15.3%)	120 (40.8%)	174 (59.2%)	0.750	0.918	0.540	1.559
	Primary	528 (27.5%)	230 (43.6%)	298 (56.4%)	0.710	0.914	0.568	1.470
	Secondary	786 (40.9%)	314 (39.9%)	472 (60.1%)	0.447	0.847	0.553	1.299
	Tertiary	312 (16.2%)	134 (42.9%)	178 (57.1%)	–	1	–	–
WHO clinical stage	Stage I	246 (12.8%)	85 (34.6%)	161 (65.4%)	0.210	0.724	0.437	1.200
	Stage II	336 (17.5%)	146 (43.5%)	190 (56.5%)	0.998	1.000	0.674	1.486
	Stage III/IV	1338 (69.7%)	567 (42.4%)	771 (57.6%)	–	1	–	–
Baseline CD 4 count	< 200 cell/μl	480 (25.0%)	177 (36.9%)	303 (63.1%)	0.072	0.737	0.528	1.028
	> 200 cell/μl	1440 (75.0%)	621(43.1%)	819 (56.9%)	–	1	–	–
BMI at baseline	Under weight	582 (30.3%)	266 (45.7%)	316 (54.3%)	0.430	1.219	0.746	1.991
	Normal	1026 (53.4%)	405 (39.5%)	621 (60.5%)	0.665	0.908	0.588	1.403
	Over weight	312 (16.2%)	127 (40.7%)	185 (59.3%)	–	1	–	–
Opportunistic infection	No	1668 (86.9%)	693 (41.5%)	975 (58.5%)	0.964	1.011	0.638	1.602
	Yes	252 (13.1%)	105 (41.7%)	147 (58.3%)	–	1	–	–
ART regimen type	AZT + 3TC + NVP	1272 (66.2%)	533 (41.9%)	739 (58.1%)	0.046	5.873	1.028	33.550
	AZT + 3TC + EFV	570 (29.7%)	234 (41.1%)	336 (58.9%)	0.055	5.566	0.962	32.198
	AZT + 3TC + ATV/r	66 (3.4%)	29 (43.9%)	37 (56.1%)	0.062	6.273	0.911	43.181
	AZT + 3TC + NVP/r	12 (0.6%)	2 (16.7%)	10 (83.3%)	–	1	–	–
Time of visit (Month)	Base line visit	320 (16.7%)	160 (50.0%)	160 (50.0%)	0.001	1.569	1.196	2.058
	6 month	320 (16.7%)	141 (44.1%)	179 (55.9%)	0.132	1.228	0.940	1.603
	12 month	320 (16.7%)	114 (35.6%)	206 (64.4%)	0.289	0.856	0.642	1.141
	18 month	320 (16.7%)	128 (40.0%)	192 (60.0%)	0.789	1.037	0.794	1.354
	24 month	320 (16.7%)	130 (40.6%)	190 (59.4%)	0.618	1.067	0.827	1.376
	30 month	320 (16.7%)	125 (39.1%)	195 (60.9%)	–	1	–	–

Univariable logistic regression analysis was done to determine the association of independent variables with the dependent variable at the end of the follow up period. Sex, age, residence, OPIs, ART regimen combination, CD4 count, social drug use, educational status and WHO clinical stage were analyzed. However, only sex, social drug use, WHO clinical stage and presence of baseline anemia have a p-value of less than or equals to 0.2 in univariable binary logistic regression analysis. These variables have been fitted to the final multivariable logistic regression model, but only sex, ART regimen combination and time of visit were remained statistically significant factors with a P-value of less than 0.05 (Additional file 2: Table S1).

### Incident anemia

Among a total of 320 patients assessed, 160 (50.0%) were free of anemia at the baseline of AZT based HAART therapy initiation. These patients were followed for 30 months with a median of 22 months (IQR = 11.5–24.5). Of them, 114 (71.3%) were females and 41.3% were within 35–45 years age category. About 51 (31.9%) of them were within 18–35 years age range. Most of them, 123 (76.9%) were urban residents. Around half 87 (54.8%) of the patients were on WHO clinical stage III and only 41 (25.6%) of them was underweight with BMI less than 18.5. The incident anemia after AZT based HAART treatment initiation was assessed by controlling the analysis of the participants’ data who were non-anemic at baseline. Incident anemia at 6, 12, 18, 24, and 30 months of follow-up were recorded in 25% (40/160), 27% (32/120), 14% (12/88), 14% (11/76) and 29% (11/38) of participants, respectively. Therefore by the end of the 30 months follow up, 106 (66.2%) of develop anemia and 54 (33.8%) were censored out 160 AZT containing HAART experienced HIV adult patients developed anemia within the observation period of two and half years (30 months). This corresponds to a cumulative incidence for this period was around 663 per 1000 patients, or an average of 265 per 1000 patients per year. The estimated survival (not developing anemia) at 6, 12, 18, 24 and 30 months were 75%, 73.0%, 86.0%, 86.0% and 71.0%, respectively (Table 6).

**Table 6:** Incidence of AZT induced anaemia in relation to duration of therapy among patients receiving HAART at University of Comprehensive specialized referral Hospital ART clinic, January 2016 to February 2018

Interval start time (Months)	Number of entering interval	Number of exposed to risk	Number of terminal events	Proportion of terminating (%)	Proportion of surviving
0	160	160.000	0	0	1.00
6	160	160.000	40	25	0.75
12	120	120.000	32	27	0.73
18	88	88.000	12	14	0.86
24	76	76.000	11	14	0.86
30	65	38.000	11	29	0.71

## Discussion

In this study, we investigated the prevalence and associated factors of anemia in a retrospective cohort of HIV/AIDS adult patients on AZT based HAART treatment over 30 months. A large proportion of participant patients were anemic at baseline and subsequent follow-up time points. Before HAART was initiated, mild, moderate and severe anemia were present in 95 (29.7%), 61 (19.1%) and 4 (1.3%) of subjects, respectively. After 30 months of HAART therapy, the overall prevalence of anemia declined to 41.6%. This indicated that, after HAART initiation the prevalence of anemia significantly decreased from 50% at baseline to 39.1% at 30 months. This was also supported by a study conducted in Zewditu Hospital, Ethiopia [1]. There was also a consistent decrease in the prevalence of mild, moderate and severe anemia after treatment with HAART. After 30 months of HAART therapy, mild, moderate and severe anemia were present in 81 (25.3%), 42 (13.1%) and 2 (0.6%) of subjects, respectively [32]. This finding signifies that anemia among AZT based HAART experienced adult HIV patients were highly affected by this disease. According to the WHO classification of the public health importance of anemia, this magnitude signifies that anemia was a moderate public health problem among the study participants [31].

The overall prevalence of anemia in our study was 41.6% (95% CI 39.4–43.8). However, the prevalence of anemia in this study was much higher than previous studies conducted in different parts of Ethiopia; 20.7% [33] and 25% [30], 22.6% [34], and 23% [35] in Gondar, Mizan-Aman and Debre-Tabor, respectively. Variation in prevalence of anemia might be due to differences in geographical variation and differences in life style across the regions.

Our finding was also higher than similar study findings conducted abroad Ethiopia such as Cambodia 11.8% [36], Banaras Hindu University 16.2% [37], Odisha, India, 14.6% [38] and Indonesia, 29.6% [39]. The reason for this difference might be due to variation in the study population with respect to their clinical stage, sociodemographic factor and difference in their life style of study population [30] or it could be due to differences in their study design short duration follow up of their study.

However, the prevalence of anemia in our study was lower than in Goma, Democratic Republic of Congo, 69% [40] and Gujarat, western India, 75% [41]. Geographical variation, socio-economic difference, life style and living standards, knowledge and attitude towards the disease might be the reason for the discrepancy of prevalence of anemia among the study population. Also the sample size used for the research might be the possible reason for the difference in the prevalence of anemia. Additionally, the general magnitude/prevalence of HIV in Ethiopia is lower than other settings in East Africa or low and middle income countries globally [42]. Therefore, it could be the reason for the lower prevalence of anemia among the study participants in our study. The other possible explanation for the anemia prevalence difference might be due to the improved coverage (73%) of free ART program that helped hundreds of thousands of ART needy people living with HIV in Ethiopia enrolled in the HAART treatment program [43].

Furthermore, our result showed that microcytic anemia followed by macrocytic anemia was found to be a common type of anemia. The general characteristic of anemia manifested in this study was dimorphic population of RBCs. This might be due to the deficiencies of iron and folic acid caused by either dietary deficiency or jejunal pathology [23] or due to malabsorption of vitamin B12 deficiency in the ileum or from gastric pathology caused by an array of infections or other conditions that affect the gastric mucosa in HIV-infected patients [24]. The microcytic anemia which is the most common type of anemia observed in this study might be due to anemia of chronic disease with low iron levels, iron deficiency, intestinal malabsorption, as well as poor intake [44].

Normocytic anemia in HIV infection and observed in a mixed nutritional problem of iron deficiency and folate or vitamin B12 deficiency, bone marrow infiltrative disease or opportunistic infection of the marrow by MAC, a destructive process such as hemolytic anemia, suppression of marrow by virus or therapy [44].

The other possible cause of the raised prevalence of macrocytic polychromic anemia might be due to the effect of drug therapy which is the common cause of megaloblastic or macrocytic anemia in HIV infected patients. Vitamin B12 and folate deficiency and liver disease also cause macrocytic anemia [44].

The 21 (16.8%) patients had an elevated RDW value in which commonly observed in HIV infected individual. Peripheral RBC changes such as anisocytosis, poikilocytosis, rouleaux formation, increased background staining are commonly observed in such patients [44].

WHO clinical stage has significant association with anemia prevalence in this study. WHO AIDS clinical stage II and III/IV were 1.82 times and 2.16 times more likely to be anemic than stage I, respectively. This was in line with a study conducted in Ethiopia [1], Bali Indonesia. This might be due to the presence of secondary infections [39].

Additionally, low baseline Hgb level was found the risk factor for developing anemia. Those anemic individuals at the baseline were 4 times more likely to develop anemia than the counterparts. Baseline anemia decreased survival, and increased disease progression in patients with HIV/AIDS. This was supported by different retrospective studies [32, 45]. The fact that anemia is a reduced level of Hgb, it is not surprising to see patients with lower value of baseline Hgb level developed anemia. Low level of Hgb supported by the additive effect of AZT for anemia due to suppression of bone marrow precursor cells [34]. AZT is associated with myelotoxity that often presents with anemia. Intolerance to AZT induced gastrointestinal problems, are important barriers to adherence unless appropriate measures are taken. Anaemia and neutropaenia are the major types of toxicity of AZT use particularly in those patients with baseline anaemia or neutropaenia, CD4 count ≤ 200 cells/mm^3^. Myopathy, lipoatrophy or lipodystrophy, Lactic acidosis or severe hepatomegaly with steatosis are additional types of AZT toxicity among those with the following risk factors; BMI > 25 (or body weight > 75 kg), and prolonged exposure to nucleoside analogues. Additionally, AZT is associated with a risk of hematological toxicity, and measuring hemoglobin is recommended before initiating ART and monthly at least for the next three months. Therefore, patients with HIV/ADIS with severe anemia at baseline should start with the other more safer types (preferred) of first-line regimens [(TDF + 3TC + DTG (FDC) or (TDF + 3TC + EFV (FDC)] [3]. The adverse event that has been occurred in greater than 40% of our study participants was anemia. Therefore, switching to other safer ART drugs like combination is very crucial [46]. Male study participants were more at high risk of developing anemia than female participants. Males were more than 1.5 times more likely to develop anemia than females. This was in agreement with some studies [1]. However, it was different from many previous studies that reported a high prevalence of anemia in females compared to male HIV infected patients were significantly associated with anemia.

HIV/AIDS patients on AZT based HAART treatment experience the highest burden of anemia at the early phases of the therapy. At the first 6 months of therapy, they were 1.6 times more at risk of anemia than the other subsequent phases. This was supported by the Central Statistics of the Ethiopia [47] and Ferede G. et al. [48]. These two studies confirmed that, anemia mostly occur within 2–48 weeks of HAART treatment initiation, especially in the first 24 weeks.

AZT drug combination was another factor found associated with the prevalence of anemia in this study. Those patients on treatment of AZT + 3TC + NVP combination were 5.9 times at risk of developing anemia than the others. Although AZT + 3TC + NVP was the recommended HAART combination used in most of the hospitals, it was in contrast to our finding and the study from Ethiopia [1], Uganda [49] and Thailand [25].

In this retrospective cohort study, 106 of 160 AZT containing HAART experienced HIV adult patients developed anemia within the observation period of two and half years (30 months). Incidence of anemia this period of study was around 663 per 1000 patients, or an average of 265 per 1000 patients per year. This finding was lower than study conducted in St. Paul’s and ALERT hospital Ethiopia, 353 per 1000 person years [50].

The imitation of this study was lack of relevant information like nutritional and socio economic status of the study participant since the information were collected from medical records retrospectively. Additionally, all patients on ART were not included in the research due to the resource limitations.

## Conclusion

Anemia among AZT containing HAART experienced adult HIV patients has been found a significant adverse event. Therefore, switching to other safer ART drugs is very crucial. Prevalence of anemia has significant association with WHO clinical stage and baseline anemia status of these patients. Therefore, preventive strategies towards these factors are necessary to reduce the magnitude of anemia the study population. There is a need to strengthen the intervention to prevent anemia. Counseling and improvement in their knowledge and attitude towards anemia might important. Further studies that address the nutritional and socio economic status of the study participant should be conducted. Further large scale studies should be conducted using a large sample size and including the assessment of all, red cell morphology, serum micronutrient level.

## Supplementary Information

**Additional file 1.** GEE Analysis Results.

**Additional file 2: Table S1.** Magnitude of anemia and Predictors of anemia among AZT based HAART experienced adult HIV patients at 30 months of HAART initiation at University of Gondar Comprehensive Specialized referral Hospital ART clinic, January 2016, to February 2018.

## Data Availability

The datasets used and/or analyzed during the current study are available from the corresponding author and it can be accessed based on reasonable request.

## Abbreviations

ADR: Adverse reaction
AIDS: Acquired immunodeficiency syndrome
ART: Antiretroviral therapy
AZT: Zidovudine
BMI: Body mass index
CD: Cluster of differentiation
CI: Confidence interval
COR: Crudes odds ratio
DNA: Deoxyribonucleic acid
HAART: Highly active antiretroviral therapy
Hgb: Hemoglobin
HIV: Human immunodeficiency virus
MCH: Mean cell hemoglobin
MCHC: Mean cell hemoglobin concentration
MCV: Mean cell volume
OIs: Opportunistic infections
RBC: Red blood cell
RDW: Red cell distribution width
RNA: Ribonucleic acid
SSA: Sub-Saharan Africa
SPSS: Statistical package for social sciences
WHO: World Health Organization
