# Supplementary material for: Magnitude and associated factors of anemia among AZT based HAART experienced adult HIV patients at University of Gondar Comprehensive Specialized Referral Hospital, Northwest, Ethiopia, 2019: a retrospective cohort study
Source: BMC Infect Dis. 2021 Sep 28;21:1016. doi: 10.1186/s12879-021-06712-5 (PMC8480035; doi:10.1186/s12879-021-06712-5)
Supplement: Supplementary file 1 — Additional file 1. GEE Analysis Results. [file 12879_2021_6712_MOESM1_ESM.docx]

| **GEE Analysis Results**  **Figure 1: Model Information** | | |
| --- | --- | --- |
| Dependent Variable | | Anemia status^a^ |
| Probability Distribution | | Binomial |
| Link Function | | Logit |
| Subject Effect | 1 | ID |
| Working Correlation Matrix Structure | | AR(1) |
| a. The procedure models Anemic as the response, treating Non anemic as the reference category. | | |

| **Figure 2: Correlated Data Summary** | | | | |  |
| --- | --- | --- | --- | --- | --- |
| Number of Levels | | Subject Effect | ID | 319 |  |
| Number of Subjects | | | | 319 |  |
| Number of Measurements per Subject | | Minimum | | 6 |  |
|  |  | Maximum | | 12 |  |
| Correlation Matrix Dimension | | | | 12 |  |
| **Figure 3: Goodness of Fit^a^** | | | | | |
|  | Value | | | | |
| Quasi Likelihood under Independence Model Criterion (QIC)^b^ | 2629.916 | | | | |
| Corrected Quasi Likelihood under Independence Model Criterion (QICC)^b^ | 2582.349 | | | | |
| Dependent Variable: Anemia status  Model: (Intercept), Sex, Residence, Educational status, Baseline WHO clinical stage, Social drug use, OPI, ART Regimen at 30month, Age category, Baseline BMI, Baseline CD4 count, Visit | | | | | |
| a. Information criteria are in small-is-better form. | | | | | |
| b. Computed using the kernel of the log quasi-likelihood function. | | | | | |

| **Figure 4: Categorical Variable Information** | | | | |
| --- | --- | --- | --- | --- |
|  | | | N | Percent |
| Dependent Variable | Anemia status | Anemic | 798 | 41.6% |
|  |  | Non-anemic | 1122 | 58.4% |
|  |  | Total | 1920 | 100.0% |
| Factor | Sex | Male | 732 | 38.1% |
|  |  | Female | 1188 | 61.9% |
|  |  | Total | 1920 | 100.0% |
|  | Residence | Rural | 498 | 25.9% |
|  |  | Urban | 1422 | 74.1% |
|  |  | Total | 1920 | 100.0% |
|  | Educational Statue | No formal education | 294 | 15.3% |
|  |  | Primary education | 528 | 27.5% |
|  |  | Secondary education | 786 | 40.9% |
|  |  | Tertiary education | 312 | 16.2% |
|  |  | Total | 1920 | 100.0% |
|  | Base line WHO clinical stage | Stage I | 246 | 12.8% |
|  |  | Stage II | 336 | 17.5% |
|  |  | Stage III | 1338 | 69.7% |
|  |  | Total | 1920 | 100.0% |
|  | ART Regimen at 30 month | AZT+3TC+NVP combination | 1272 | 66.2% |
|  |  | AZT+3TC+EFV combination | 570 | 29.7% |
|  |  | AZT+3TC+ATV/r combination | 66 | 3.4% |
|  |  | AZT+3TC+NVP/r combination | 12 | 0.6% |
|  |  | Total | 1920 | 100.0% |
|  | Age category | <35 years | 660 | 34.4% |
|  |  | 35-45 years | 798 | 41.6% |
|  |  | > 45 years | 462 | 24.1% |
|  |  | Total | 1920 | 100.0% |
|  | Categorized BMI at baseline | under weight | 582 | 30.3% |
|  |  | Normal | 1026 | 53.4% |
|  |  | Over weight | 312 | 16.2% |
|  |  | Total | 1920 | 100.0% |
|  | Categorized baseline CD4 count | CD4 count <200 cells/ul | 480 | 25.0% |
|  |  | CD4 count> 200 cells/ul | 1440 | 75.0% |
|  |  | Total | 1920 | 100.0% |
|  | Duration of treatment | Base line | 320 | 16.7% |
|  |  | 6 month | 320 | 16.7% |
|  |  | 12 month | 320 | 16.7% |
|  |  | 18 month | 320 | 16.7% |
|  |  | 24 month | 320 | 16.7% |
|  |  | 30 month | 320 | 16.7% |
|  |  | Total | 1920 | 100.0% |

| **Supplementary Table2: The parameter estimates of the GEE model** | | | | | | | | | | |
| --- | --- | --- | --- | --- | --- | --- | --- | --- | --- | --- |
| Parameter | B | Std. Error | 95% Wald Confidence Interval | | Hypothesis Test | | | Exp(B) | 95% Wald Confidence Interval for Exp(B) | |
|  |  |  | Lower | Upper | Wald Chi-Square | df | Sig. |  | Lower | Upper |
| (Intercept) | -2.905 | .9533 | -4.774 | -1.037 | 9.288 | 1 | .002 | .055 | .008 | .355 |
| [Sex=1] | .423 | .1662 | .097 | .749 | 6.485 | 1 | **0.011** | **1.527** | **1.102** | **2.114** |
| [Sex=2] | 0^a^ | . | . | . | . | . | . | 1 | . | . |
| [Residence=1] | .328 | .1714 | -.008 | .664 | 3.665 | 1 | .056 | 1.388 | .992 | 1.943 |
| [Residence=2] | 0^a^ | . | . | . | . | . | . | 1 | . | . |
| [Educationalstatus=0] | -.086 | .2705 | -.616 | .444 | .101 | 1 | .750 | .918 | .540 | 1.559 |
| [Educationalstatus=1] | -.090 | .2427 | -.566 | .386 | .138 | 1 | .710 | .914 | .568 | 1.470 |
| [Educationalstatus=2] | -.165 | .2178 | -.592 | .261 | .577 | 1 | .447 | .847 | .553 | 1.299 |
| [Educationalstatus=3] | 0^a^ | . | . | . | . | . | . | 1 | . | . |
| [BaselineWHOclinicalstage=1] | -.323 | .2574 | -.827 | .182 | 1.570 | 1 | .210 | .724 | .437 | 1.200 |
| [BaselineWHOclinicalstage=2] | .000 | .2018 | -.395 | .396 | .000 | 1 | .998 | 1.000 | .674 | 1.486 |
| [BaselineWHOclinicalstage=3] | 0^a^ | . | . | . | . | . | . | 1 | . | . |
| [Socialdruguse=0] | .581 | .3154 | -.037 | 1.199 | 3.392 | 1 | .066 | 1.788 | .963 | 3.317 |
| [Socialdruguse=1] | 0^a^ | . | . | . | . | . | . | 1 | . | . |
| [OPI=0] | .011 | .2350 | -.450 | .471 | .002 | 1 | .964 | 1.011 | .638 | 1.602 |
| [OPI=1] | 0^a^ | . | . | . | . | . | . | 1 | . | . |
| [ARTRegimen =1] | 1.770 | .8891 | .028 | 3.513 | 3.964 | 1 | **0.046** | **5.873** | **1.028** | **33.550** |
| [ARTRegimen=2] | 1.717 | .8956 | -.039 | 3.472 | 3.674 | 1 | .055 | 5.566 | .962 | 32.198 |
| [ARTRegimen=3] | 1.836 | .9842 | -.093 | 3.765 | 3.481 | 1 | .062 | 6.273 | .911 | 43.181 |
| [ARTRegimen=4] | 0^a^ | . | . | . | . | . | . | 1 | . | . |
| [Age_catagory=1.00] | .184 | .2219 | -.251 | .619 | .690 | 1 | .406 | 1.202 | .778 | 1.858 |
| [Age_catagory=2.00] | .128 | .2126 | -.289 | .544 | .360 | 1 | .549 | 1.136 | .749 | 1.723 |
| [Age_catagory=3.00] | 0^a^ | . | . | . | . | . | . | 1 | . | . |
| BMI_at_baseline=1.00] | .198 | .2506 | -.294 | .689 | .622 | 1 | .430 | 1.219 | .746 | 1.991 |
| [BMI_at_baseline=2.00] | -.096 | .2216 | -.530 | .338 | .188 | 1 | .665 | .908 | .588 | 1.403 |
| BMI_at_baseline=3.00] | 0^a^ | . | . | . | . | . | . | 1 | . | . |
| [Baseline_CD4_count=1.00] | -.305 | .1698 | -.638 | .028 | 3.229 | 1 | .072 | .737 | .528 | 1.028 |
| [Baseline_CD4_count=2.00] | 0^a^ | . | . | . | . | . | . | 1 | . | . |
| [Visit=1] | .450 | .1386 | .179 | .722 | 10.556 | 1 | **0.001** | **1.569** | **1.196** | **2.058** |
| [Visit=2] | .205 | .1363 | -.062 | .472 | 2.263 | 1 | .132 | 1.228 | .940 | 1.603 |
| [Visit=3] | -.156 | .1467 | -.443 | .132 | 1.126 | 1 | .289 | .856 | .642 | 1.141 |
| [Visit=4] | .036 | .1359 | -.230 | .303 | .071 | 1 | .789 | 1.037 | .794 | 1.354 |
| [Visit=5] | .065 | .1298 | -.190 | .319 | .248 | 1 | .618 | 1.067 | .827 | 1.376 |
| [Visit=6] | 0^a^ | . | . | . | . | . | . | 1 | . | . |
| (Scale) | 1.016 |  |  |  |  |  |  |  |  |  |
| Dependent Variable: Anemia status  Model: (Intercept), Sex, Residence, Educationalstatus, BaselineWHOclinicalstage, Socialdruguse, OPI, ARTRegimenat30month, Age_catagory, Cat_BMI_at_baseline, Cat_baseline_CD4_count, Visit | | | | | | | | | | |
| a. Set to zero because this parameter is redundant. | | | | | | | | | | |
