# Supplementary material for: Magnitude and associated factors of anemia among AZT based HAART experienced adult HIV patients at University of Gondar Comprehensive Specialized Referral Hospital, Northwest, Ethiopia, 2019: a retrospective cohort study
Source: BMC Infect Dis. 2021 Sep 28;21:1016. doi: 10.1186/s12879-021-06712-5 (PMC8480035; doi:10.1186/s12879-021-06712-5)
Supplement: Supplementary file 2 — Additional file 2: Table S1. Magnitude of anemia and Predictors of anemia among AZT based HAART experienced adult HIV patients at 30 months of HAART initiation at University of Gondar Comprehensive Specialized referral Hospital ART clinic, January 2016, to February 2018. [file 12879_2021_6712_MOESM2_ESM.docx]

**Supplementary Table 1.** Magnitude of anemia and Predictors of anemia among AZT based HAART experienced adult HIV patients at 30 months of HAART initiation at University of Gondar Comprehensive Specialized referral Hospital ART clinic, January 2016, to February 2018.

|  | Category | Anemia status | | P-value | COR( 95% CI) | AOR( 95% CI) |
| --- | --- | --- | --- | --- | --- | --- |
|  |  | Anemic | Non anemic |  |  |  |
| Age category | <35 | 43(39 %) | 67(61%) | 1 |  |  |
|  | 35-45 | 54(41%) | 79(59%) | 0.811 | 0.94(0.56-1.57) |  |
|  | >45 | 28(36%) | 49(64%) | 0.703 | 1.123(0.615-2.050) |  |
| Sex | Male | 45(37%) | 77(63%) | 1 |  |  |
|  | Female | 80(40%) | 118(60%) | 0.531 | 1.16(0.75-1.85) |  |
| Residence | Urban | 38(20 %) | 150(80 %) | 0.146 | 1.46(0.88-2.42) | 1.37 (0.80 – 2.34) |
|  | Rural | 87(66%) | 45(34%) | 1 |  |  |
| Educational status | No education | 16(33%) | 33(67%) | 0.682 | 1.19(0.052-2.70) |  |
|  | Primary | 38(43%) | 50(57%) | 0.440 | 0.76(0.37-1.53) |  |
|  | Secondary | 52(40%) | 79(60%) | 0.693 | 0.89(0.45-1.67) |  |
|  | Tertiary | 19 (37%) | 33(63) | 1 |  |  |
| WHO clinical stage | I | 65(46%) | 76(54%) | 1 |  |  |
|  | II | 44(35%) | 81(65%) | 0.072 | 1.57(0.96-2.58) | **1.75 (1.03 – 2.96)*** |
|  | III | 16(31%) | 38(69%) | **0.039** | **2.03(1.04-3.97)** | **2.09 (1.11 – 4.22)**** |
| BMI | Under weight | 30(45%) | 36(55%) | 0.477 | 0.78(0.40-0.54) |  |
|  | Normal | 67(37%) | 116(33%) | 0.676 | 1.13(0.64-1.98) |  |
|  | Over weight | 28(39%) | 43(61%) | 1 |  |  |
| ART regimen | AZT+3TC+NVP | 87(41%) | 125 (59%) | 0.467 | 0.69(0.19-2.14) |  |
|  | AZT+3TC+EFV | 34(36%) | 61(64%) | 0.723 | 0.80(0.23-2.78) |  |
|  | AZT+3TC+ATV/r | 4(36%) | 7 (64%) | 1 |  |  |
| CD4 count | CD4 count < 200 cells/ul | 10 (44%) | 13 (56%) | 0.653 | 0.82(0.35-1.94) |  |
|  | CD4 count > 200 cells/ul | 115(39%) | 182 (61%) | 1 |  |  |
| Social drug use | Yes | 4 (20%) | 16 (80%) | 0.083 | 2.70(0.88-8.28) | 2.10 ( 0.63 – 6.96) |
|  | No | 121(40 %) | 179 (60 %) | 1 |  |  |
| OPIs | Yes | 16 (38%) | 26 (62 %) | 0.890 | 1.05(0.54-2.04) |  |
|  | No | 109(39%) | 169(61 %) | 1 |  |  |
| Baseline  Anemia | Yes | 88 (55%) | 72 (45%) | **< 0.001** | **4.06(2.51 – 6.56)** | **3.98 (2.45 –6.48)***** |
|  | No | 37 (23%) | 123 (77 %) | 1 |  |  |

COR= crude odds ratio AOR= adjusted odds ratio

*** P-value = 0.038 ** p-value=0.024 *** p-value <0.001**
